# Preliminary Evaluation of the Nutraceutical Properties in Monovarietal Extra-Virgin Olive Oils and Monitoring Their Stability During Storage

**DOI:** 10.3390/molecules30153143

**Published:** 2025-07-26

**Authors:** Lina Cossignani, Ornella Calderini, Antonello Marinotti, Emiliano Orrico, Andrea Domesi, Luisa Massaccesi, Mirko Cucina, Marina Bufacchi

**Affiliations:** 1 Section of Food, Biochemical, Physiological and Nutritional Sciences, Department of Pharmaceutical Sciences, University of Perugia, 06126 Perugia, Italy; 2 Institute of Biosciences and Bioresources, National Research Council, 06128 Perugia, Italy; ornella.calderini@cnr.it; 3 Institute for Agricultural and Forest Systems in the Mediterranean, National Research Council, 06128 Perugia, Italy; antonellomarinotti@cnr.it (A.M.); emiliano.orrico@cnr.it (E.O.); andrea.domesi@cnr.it (A.D.); luisa.massaccesi@cnr.it (L.M.); mirko.cucina@cnr.it (M.C.)

**Keywords:** extra-virgin olive oil, bioactive compounds, storage, chemical stability, chemometric analysis

## Abstract

In this paper, an in-depth characterization of the composition of extra-virgin olive oil (EVOO) from different cultivars was performed, with the aim of obtaining the fingerprint profile of bioactive constituents and studying the oxidative stability of the samples, both by an accelerated stability test and after four months of storage at room temperature. Among the investigated cultivars, some were typical of Umbria (Central Italy), namely Moraiolo, Frantoio, and Dolce Agogia, others of Apulia (Southern Italy), Coratina, Peranzana, and Bella di Cerignola, and others were typical Spanish cultivars cultivated in Umbria (Arbequina and Arbosana). The comparison of the chemical parameters among oils from the different cultivars allowed for their discrimination by multivariate statistical analysis. Some phenolic compounds were mainly responsible for the sample group’s differentiation, with the oils from the Spanish cultivars clearly distinguished from the Umbrian and Apulian sample groups. The processing of the results by chemometric analysis during oil storage and stability tests again allowed the discrimination of the samples analyzed at different storage times. This study contributes to increasing knowledge on olive oils—chemical and nutraceutical properties from specific cultivars, particularly some less studied so far, such as the Bella di Cerignola cultivar, and their changes in their nutraceutical properties during storage.

## 1. Introduction

Extra-virgin olive oil (EVOO), an integral component of the Mediterranean diet, is well known for its organoleptic and nutritional characteristics, as well as its health-promoting properties [1]. A large body of evidence has demonstrated that EVOO consumption has protective effects on chronic conditions, among which are cardiovascular diseases [2], metabolic syndrome [3], neurodegenerative disorders [4], and cancer [5]. These beneficial effects have been largely attributed to the composition of EVOO, characterized by a high concentration of oleic acid, along with several valuable minor components, including phenolic compounds, sterols, α-tocopherol, pigments such as chlorophylls and carotenoids (β-carotene and lutein), hydrocarbons, and volatile compounds [6,7]. The influence of several factors on EVOO composition, including variety, harvesting time, agronomic, environmental, and technological conditions, has been widely reported [7]. Based on these premises, the increasing demand for EVOO on the global market is justified by its unique chemical, sensory, and health characteristics. The high economic value of this premium food product makes it susceptible to adulteration with lower-quality oils. Strict regulations have been adopted by the European Union (EU) with Regulation (EEC) 2568/91 (1991) and following amendments [8], as well as the International Olive Oil Council (IOC) trade standard [9], to guarantee EVOO quality and authenticity.

Besides the addition of adulterating oils, the quality and overall health benefits of EVOO can decrease over time due to oxidation, exposure to light, and improper storage conditions. Therefore, monitoring the shelf life of olive oil through chemical analysis is crucial to preserve the chemical integrity of the oil and ensure high-quality products for consumers [7].

Chemical analysis provides objective metrics for evaluating olive oil quality, including parameters such as free fatty acid content and peroxide value, markers of hydrolysis and primary oxidation, respectively. Accelerated shelf life testing procedures were applied to speed up the oxidation process and develop shelf life predictive models. However, it has been reported that extrapolation from accelerated tests at high temperatures could lead to inaccurate predictions of the real shelf life of olive oil [10]. For these reasons, accelerated tests at mild temperatures (lower than 60 °C) were performed in parallel with shelf life tests at ambient conditions [11]. For example, some researchers performed an accelerated test in the dark at mild temperatures (from 40 to 60 °C) and found that K232 showed an excellent correlation with the loss of unsaturated fatty acids [12]. The same authors, more recently, focused on EVOO minor components, such as phenols, tocopherol, and pigments, to investigate a shelf life predictive model [13]. More recently, extra-virgin olive oils were stored at increasing temperatures (from 25 to 60 °C) in the dark under reduced oxygen content [14]. The authors concluded that K270 and % phyropheophytin a were good indicators of EVOO stability during storage.

The role of natural antioxidant compounds, i.e., phenols and tocopherols, in the shelf life and quality of EVOO has been extensively investigated. It is widely known that phenolic compounds are critical for both the health benefits of olive oil and its sensory properties. Research demonstrates that as olive oil ages, the concentration of these beneficial compounds can diminish, leading to reduced health efficacy and deterioration of sensory attributes, which can ultimately affect consumer acceptance [15]. Castillo-Luna and colleagues investigated the influence of the initial phenolic content on the decrease in phenolic content in EVOO stored at room temperature for 12 months and found that the samples richer in oleacein and oleocanthal showed a larger loss of phenolic content than other oil samples [16].

Furthermore, the measurement of tocopherol content during EVOO storage is of great importance due to its protective role against the progression of oxidation. Gagour et al. performed the chemical characterization and investigated the shelf life behavior of two Moroccan EVOO cultivars under accelerated conditions (60 °C) [17]. They observed a marked decrease in tocopherol content, more marked for “Arbequina” (62%) after 8 weeks than Moroccan Picholine (48%).

Among minor components, squalene has also been monitored during EVOO storage in simulated domestic conditions, and it remained rather stable up to six months [15]. Even under accelerated storage conditions (60 °C), squalene showed good stability, with a loss lower than 20% within the induction period [18]. Moreover, sterolic components, also responsible for the beneficial effects of olive oil consumption on human health, remained almost stable after six months of storage at room temperature in different containers [19].

This work aimed to investigate the correlation between the content of EVOO bioactive compounds and the stability during storage, such as the impact of storage in domestic conditions on the chemical and nutritional/healthy properties of different EVOO cultivars. In particular, EVOO samples from different cultivars cultivated in Umbria (Central Italy), namely, Moraiolo, Frantoio, Dolce Agogia, Arbequina, and Arbosana, and Apulia (Southern Italy), Coratina, Peranzana, and Bella di Cerignola, were studied. To the best of our knowledge, this is the first time a systematic characterization and stability study has been performed on the Apulian cultivars, especially considering the Bella di Cerignola one.

## 2. Results and Discussion

### 2.1. Characterization of Chemical Parameters in Different EVOO Cultivars

**Figure 1:**
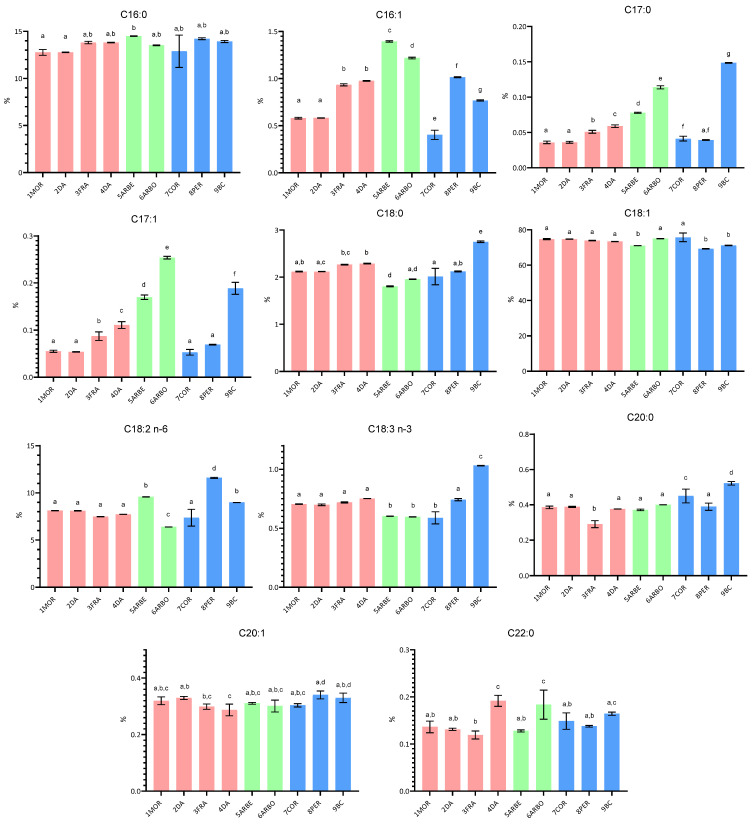
Bar graph showing the arithmetic mean and standard deviation of the percentage fatty acid content of EVOO from the selected cultivars (samples from Umbrian cultivars are indicated with red bars, those from Spanish cultivars with green bars, and those from Apulian cultivars with light-blue bars). Different superscript letters above the bars indicate significantly different values (Tukey’s test, *p* ˂ 0.05).

**Figure 2:**
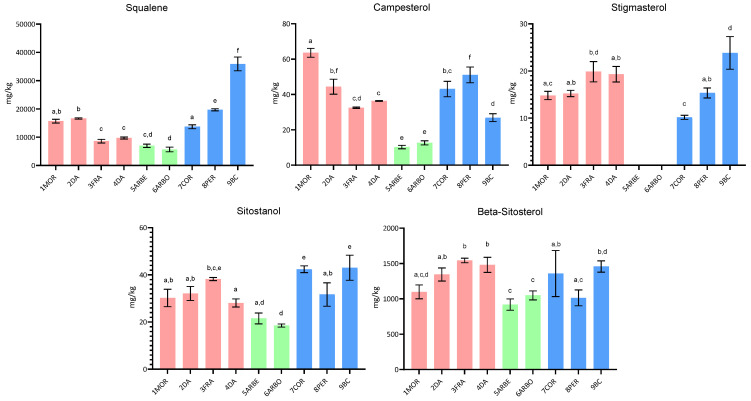
Bar graph showing the arithmetic mean and standard deviation of the content of squalene and sterol compounds in EVOO from the selected cultivars (samples from Umbrian cultivars are indicated with red bars, those from Spanish cultivars with green bars, and those from Apulian cultivars with light-blue bars). Different superscript letters above the bars indicate significantly different values (Tukey’s test, *p* ˂ 0.05).

**Figure 3:**
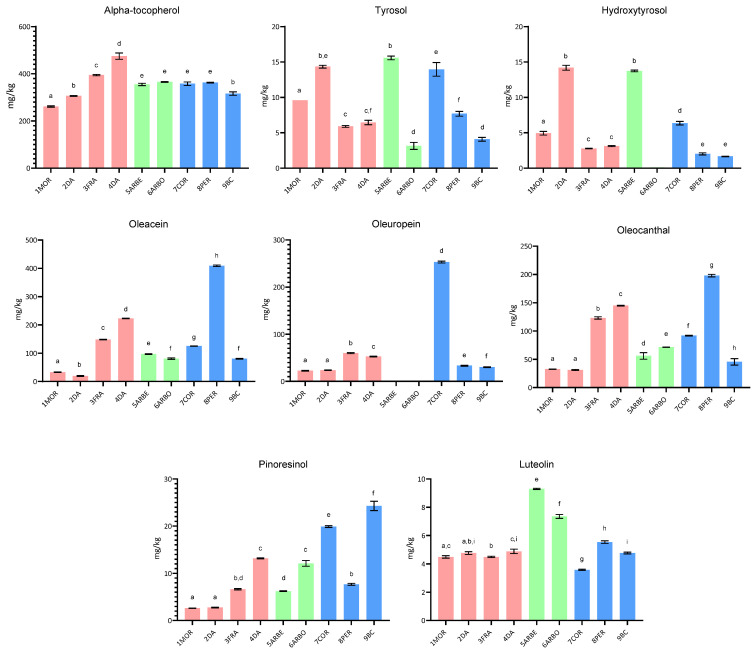
Bar graph showing the arithmetic mean and standard deviation of the content of alpha-tocopherol and phenolic compounds in EVOO from the selected cultivars (samples from Umbrian cultivars are indicated with red bars, those from Spanish cultivars with green bars, and those from Apulian cultivars with light-blue bars). Different superscript letters above the bars indicate significantly different values (Tukey’s test, *p* ˂ 0.05).

This paper investigated the compositional characteristics, the sensory properties, and the oxidative stability of EVOO samples obtained from selected cultivars, cultivated in central Italy (Umbria) and the Apulia region. In addition to some typical Umbrian and Spanish cultivars cultivated in the Umbria region, some Apulian cultivars were also studied, including the little investigated Bella di Cerignola. In Figure 1, Figure 2 and Figure 3, the fatty acid composition, the squalene and sterol content, and the content of tocopherol and phenolic compounds are reported, respectively.

#### 2.1.1. Fatty Acid Composition

The fatty acid composition shows some differences among the grouped samples (Figure 1). In particular, C16:0 and C17:0 content was lower in EVOO samples from Umbrian cultivars than the Spanish ones (*p* < 0.05), while the content of C16:1, C17:1, C18:0, and C18:3 n3 showed significant differences between the Spanish cultivars and Umbrian and Apulian ones. Lastly, C20:0 was more represented in Apulian cultivars than both Umbrian and Spanish ones (*p* < 0.05). It has been widely reported that olive oil composition from any specific cultivars is due to a very complex interaction among the genotypic potential and the environmental, agronomic, and technological factors [20]. It is noteworthy that variations in fatty acid percentage content may be due to climatic trends (year effect), in particular the period from stone hardening to the end of fruit enlargement [21]. Use of the fatty acid composition as a potential marker of the geographical and genetic origin of olive oil has been proposed, both considering the absolute content of individual components and considering some ratios between them, such as oleic/linoleic or palmitic/linoleic ratios [22]. Our results showed that EVOO from Umbrian cultivars had a higher palmitic/linoleic ratio (*p* < 0.05) than the Apulian ones, due to a lower polyunsaturated fatty acid content.

It was also observed that Arbosana EVOO had the highest monounsaturated/polyunsaturated fatty acid ratio, while Peranzana had the lowest. This is due to the lowest content of linoleic acid in the first (6.39%) and the highest in the second cultivar (11.60%). Moreover, it is also interesting to highlight that the Bella di Cerignola sample presented the highest content of alpha-linolenic acid (1.03%), which was slightly higher than the limit of 1% established by EU regulation [8]. However, in the same Regulation, it is specified that when the linolenic acid is more than 1.00 but less than or equal to 1.40, the apparent β-sitosterol/campesterol ratio has to be greater than or equal to 24. This last condition was verified for this sample, with the ratio being equal to 56.06. A similar result has been obtained by other authors, who reported a linolenic acid content of 0.94% for table olives from the Bella di Cerignola cultivar grown in Lebanon [23]. It is not easy to find literature data on the chemical composition of EVOO from this cultivar, since its drupes are not commonly used to produce oil, but rather table olives. In fact, Bella di Cerignola is a traditional variety of table olive from the Apulian region that received the protected denomination of origin (PDO) from the European Union with the full name of Bella della Daunia, variety Bella di Cerignola.

Figure 1 also shows that the Spanish varieties had significantly higher C16:1 and C17:1% and lower C18:0 and C18:3 n3 content than the other cultivars. Moreover, palmitic acid from Arbequina EVOO was the highest (14.50%) among the selected cultivars, as also reported by other authors who investigated the fatty acid content in eight different monovarietal virgin olive oils from Extremadura [24].

#### 2.1.2. Squalene and Sterol Content

Regarding the squalene and sterol content, Figure 2 shows the results obtained for EVOO from the investigated Umbrian, Spanish, and Apulian cultivars, where marked differences among samples are evident. First of all, squalene content showed significant differences among EVOO from all three grouped cultivars. The highest squalene content was measured in Apulian samples (*p* < 0.05), with an average concentration of 23,093.8 mg/kg, and an absolutely higher content in the Bella di Cerignola cultivar (35,893.4 mg/kg). On the contrary, the lowest squalene content (average 6302.3 mg/kg) was measured in the Spanish cultivars (*p* < 0.05), in particular the Arbosana cultivar (5652.9 mg/kg).

Other authors have reported that EVOO samples from Arbosana and Arbequina cultivars grown in Sicily showed a lower concentration of squalene than typical Sicilian cultivars [25]. Aresta and colleagues (2020) also reported the differences in squalene content among different monovarietal olive oils, showing higher concentrations in the Apulian Peranzana cultivar than in the Umbrian cultivars, as Moraiolo and Frantoio [26]. It has been reported that squalene content shows a wide variability in different olive cultivars grown in the same orchard in Cordoba, with the fruits harvested at the same ripening index with the same extraction process [27]. The authors were thus able to explain the high variability in squalene content by genetic factors and to hypothesize that squalene content could also be used as a valid marker for characterization and discrimination of monovarietal virgin olive oils.

The results obtained in this study also showed that sterol components had a lower content in oil from the Spanish than both Umbrian and Apulian cultivars (*p* < 0.05), which, on the contrary, were characterized by not significantly different results, with the sole exception of sitostanol (Figure 2).

It is widely known that sterols are biologically active compounds of great relevance for olive oil quality, as well as that their concentration and the proportions of particular sterols and triterpene diols are among the parameters used to verify and prove the authenticity of olive oil under the EU regulation [8]. Boskou (2006) highlighted that several factors can affect the sterol composition and content of olive oil, among which are cultivar, crop year, and degree of fruit ripeness, but also the storage time of olives before extraction and the extraction method itself [28]. Torres et al. (2022) reported the effect of olive growing environments, differing in thermal regime conditions, on squalene and sterol contents of olive fruits from Arbequina and Coratina cultivars grown in different Argentinian environments [29]. They also verified an effect of the cultivar, particularly on the contents of β-sitosterol, campesterol, and total sterols, with higher content found in Coratina than Arbequina olives, differently from the results reported in this paper for the same monovarietal olive oils. However, other authors investigated the composition of virgin olive oil from European cultivars grown in China and found that Arbequina had a lower content of sterols than Coratina, Arbosana, and Frantoio monovarietal olive oil [30].

#### 2.1.3. Content of α-Tocopherol and Phenolic Compounds

Lastly, in this work, the main EVOO antioxidant compounds, alpha-tocopherol and phenols, were determined in the monovarietal samples, since they play a key role in the healthy properties and the oxidative stability/shelf life of olive oils. The results of tocopherol content, shown in Figure 3, gave no significant differences for the Umbrian, Spanish, and Apulian cultivars (*p* > 0.05), with average values of 359.4, 360.3, and 345.7 mg/kg, respectively. However, among Umbrian cultivars, sample 4DA showed the highest content, while 1MOR showed the lowest among the samples from Umbrian cultivars and the other investigated EVOO samples.

Different tocopherol content was found in EVOO, at three different ripening stages, from the Arbequina cultivar grown in the southwest of Spain [31]. It varied from 371 to 213 mg/kg in oils obtained from green to ripe olives, reaching a value lower than that measured in this study. Regarding two Italian cultivars, Leccino and Frantoio, other authors reported fluctuating tocopherol concentrations during three years in the two cultivars, with no clear trend for distinguishing organic from conventional oils [32]. They reported a tocopherol content generally higher in EVOO from Leccino than Frantoio, with values for the latter cultivar ranging from 119 to 148 mg/kg, again lower than the value obtained in this work for Frantoio EVOO.

For Coratina olive oil, a study on changes in EVOO from Coratina grown in Apulia during a prolonged storage reported an initial value of 240 mg/kg [33], lower than that found in this study for the same Apulian cultivar (358 mg/kg).

Figure 3 also shows the results of the main phenolic compounds obtained for EVOO from the cultivars selected for this study. It is possible to observe that the concentration of these bioactive secondary metabolites greatly varies among the grouped cultivars, but also within the same cultivar group.

However, it is noteworthy that for the two simple phenolic alcohols, that is, tyrosol and hydroxytyrosol, no significant differences were obtained among the grouped cultivars (*p* > 0.05), even if a lower average value (3.34 mg/kg) was obtained for Apulian cultivars than the Umbrian (6.26 mg/kg) and the Spanish ones (6.92 mg/kg). Surprisingly, high differences were found between two Spanish cultivars, Arbequina and Arbosana, for both tyrosol and hydroxytyrosol. Higher values of the two phenolic compounds were measured in the Arbequina than in the Arbosana cultivar (*p* < 0.05), the latter cultivar presenting only a trace of hydroxytyrosol. The results obtained for the secoiridoids, oleuropein, oleacein, and oleocanthal, showed a great variability, with the highest values registered for EVOO from Apulian Peranzana and Coratina cultivars, followed by Frantoio and Dolce Agogia-DA4 samples, while the lowest were for Spanish cultivars and some Umbrian ones (Frantoio and Dolce Agogia-DA2). In particular, oleacein content differed among cultivars, Peranzana being the richest one, even if the average content was not significantly different among the grouped cultivars (*p* > 0.05). Oleuropein was instead much represented in Coratina EVOO, while detected only in trace amounts in Spanish cultivars. The average content of oleocanthal was also lowly represented in the Spanish cultivars and mostly in the Apulian ones, with no significant differences among the grouped samples (*p* > 0.05). The results obtained for the lignan pinoresinol confirmed that two Apulian cultivars, Coratina and Bella di Cerignola, were characterized by the highest concentration among the other cultivars under investigation (*p* < 0.05), while the flavone luteolin was mostly concentrated in the Spanish cultivars than in the others (*p* < 0.05). Although the number of samples in this study is limited, the results underscore notable differences among samples from different cultivars. It is important to note, however, that the phenolic content of EVOO is influenced not only by the cultivar but also by agronomic practices, environmental conditions, technological processes, and storage, all of which can affect both the qualitative and quantitative phenolic profile. In fact, after production, EVOO can be subjected to degradation processes, in particular hydrolytic and oxidative reactions that influence the phenolic profile. It has been reported that during storage, oleocanthal and oleacein can be converted into the corresponding phenolic alcohols, tyrosol, and hydroxytyrosol [34]. Extensive research has been conducted to highlight the chemical aspects and the biological properties of the EVOO phenolic fraction. Furthermore, investigations on the agronomic and technological factors affecting the qualitative and quantitative composition of these compounds in EVOO have been widely carried out. Del Coco and colleagues (2014) investigated the phenolic compounds in monovarietal EVOO samples from the Coratina cultivar and compared results with monovarietal EVOO from other Apulian cultivars and other Mediterranean countries [35]. They found high signals for oleuropein and glycoside, their aglycone and dialdehyde forms. It is well known that EVOO samples from the Coratina cultivar present a bitter flavor, mainly caused by oleuropein glucoside and its aglycone [20]. These results agree with our study’s findings, showing high oleuropein content in Coratina EVOO. Additionally, the findings of Mansouri et al. (2019), who found a higher pinoresinol content for Arbosana than Arbequina oils (7.26 and 4.55 mg/kg, respectively), agree with our results, even if higher absolute values were obtained in the present study (12.13 and 6.21 mg/kg) [36]. Concerning oleacein and oleocanthal, interesting results were obtained for EVOO from the Peranzana cultivar, higher than the results obtained by Baiano and colleagues, who studied the effect of cultivar and location on the phenolic profile of EVOO from the Apulia region [33].

#### 2.1.4. Principal Components Analysis and Linear Discriminant Analysis

A statistical approach has also been applied to the whole data matrix to extract information related to the study, using the MetaboAnalyst tool. Data autoscaling was performed before data processing to ensure a uniform variable scale. One-way analysis of variance (ANOVA) (*p* < 0.05) results, shown in Table S1, indicate 13 significant variables, among which stigmasterol, oleuropein, luteolin, campesterol, and squalene are the most significant, with values of −log10(p) from 20.24 for stigmasterol to 4.82 for squalene. Regarding the correlation analysis, Figure S1 shows coefficients higher than 0.90 for the variables oleuropein-stigmasterol, oleocanthal-oleacein, C18:3 n3-C18:0, C17:1-C17:0, and luteolin-C16:1, while higher than 0.80 for oleuropein-campesterol, stigmasterol-campesterol, tyrosol-hydroxytyrosol, oleocanthal-alpha tocopherol, C18:1 n9+n7-C20:1, C16:0-C16:1, C16:0-C20:1, C17:1-C16:1, and C17:1-luteolin. Figure S1 also shows some negative correlations, indicated by a correlation coefficient higher than −0.80, such as the pairs squalene-free acidity, oleuropein-luteolin, and hydroxytyrosol-C22:0.

**Figure 4:**
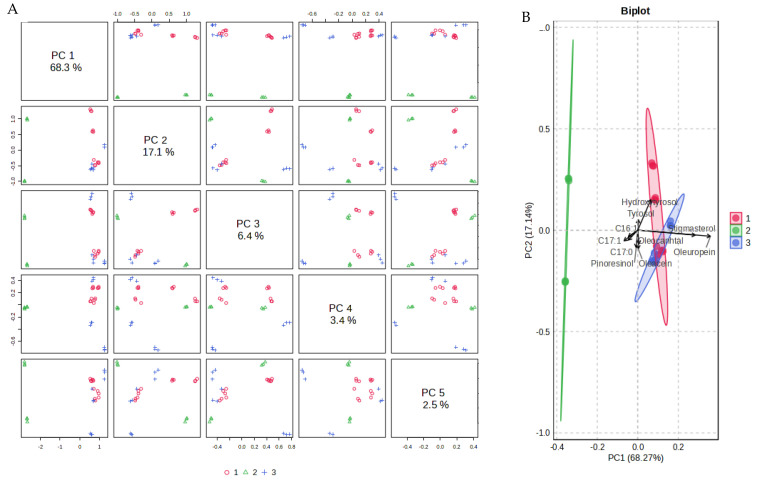
EVOO metabolite processing, T0. PCA pairwise score plots for the five Principal Components (**A**) and biplot (**B**).

Regarding multivariate analysis, at first, Principal Component Analysis (PCA) was applied to the metabolite contents as an unsupervised method for dimensionality reduction and data exploration. Figure 4A shows the pairwise score plot for the first five Principal Components (PC) with the variance explained, which provides an overview of the various separation patterns between the most important PC in the study.

It is possible to conclude that the two-dimensional solution can validly represent the relevant data structure, with 68.3 + 17.1 = 85.4% of the variance explained. The biplot, a combined plot of PCA score and loading plot in the first two PC, is reported in Figure 4B. It enables visualization of the original feature contribution in the space defined by the first two PC. The length of the lines indicates the amount of variance explained by each variable, while the closeness of the lines refers to a correlation between them. With this tool, it is possible to identify features with non-redundant information contributing to maximum variance within each PC. Figure 4B shows clear sample groupings, indicating cultivar-specific variation, with evident differences for group 2, the Spanish cultivars. Moreover, it is possible to observe that several phenolic compounds (above all oleuropein and hydroxytyrosol) and stigmasterol mostly contributed to the group classification, with some minor fatty acids (C16:1, C17:0, and C17:1) having a lower weight.

**Figure 5:**
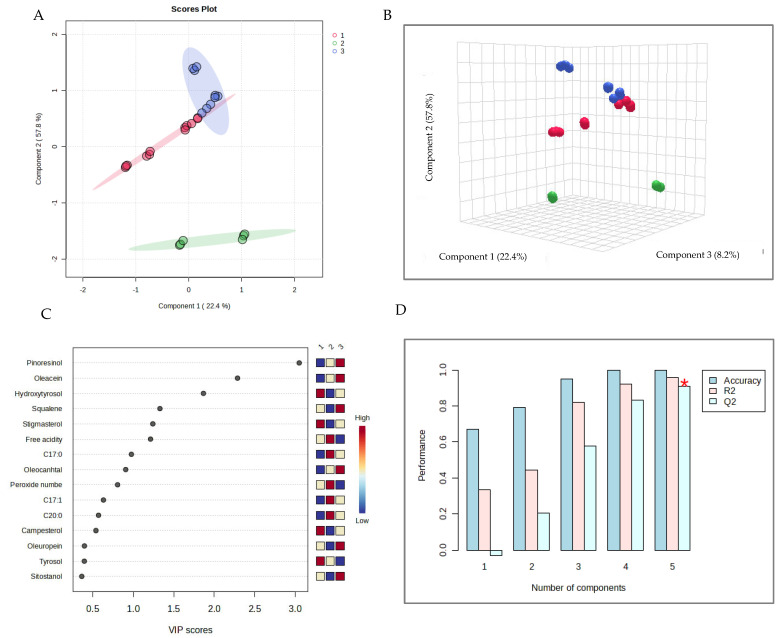
EVOO metabolite processing, T0. PLS-DA: 2D score plot (**A**); 3D score plot (**B**); variable importance in projection (**C**); cross-validation (**D**).

Subsequently, Partial Least Squares Discriminant Analysis (PLS-DA) was applied as a linear classification technique to classify EVOO samples based on the cultivar group. Figure 5A and Figure 5B show the 2D score plot, with the 95% confidence region, and the 3D score plot, respectively.

It is possible to observe that group 2, the Spanish samples, is very far from the other two groups, which also showed a good separation. The second discriminant function had the highest discriminative power, as indicated in the score plots. Variable Importance in Projection (VIP) is reported in Figure 5C, together with the colored boxes on the right, indicating the relative concentrations of the corresponding metabolite in each group under study. Pinoresinol had the highest VIP score, with an increasing concentration from sample group 1 to sample group 3. Additionally, oleacein had a high VIP score and the same content trend as pinoresinol, while hydroxytyrosol had the highest concentration in group 1 and the lowest in group 2. Moreover, cross-validation for the optimal number of components for classification was performed. The maximum number of components to search was five, and a 5-fold cross-validation method was used. Among the performance measures (Accuracy, R2, and Q2), the Q2 was chosen, as it estimates the model’s predictive ability. The results of cross-validation, shown in Figure 5D, indicate good model performance with three components, even if five was the optimal number of components for classification.

### 2.2. Sensorial Analysis and Phenolic Content

The EVOO samples have been subjected to organoleptic evaluation, as required by current Regulations [8,9]. The sensory analysis of olive oil measures negative attributes and three positive attributes. The first are the sensorial manifestations of unhealthy olive fruit, poor cultivation, harvesting practices, extraction processes, and unsuitable storage and preservation conditions. On the contrary, the positive attributes characterizing olive oil are fruitiness, bitterness, and pungency. Among these, bitterness and pungency are mainly due to the qualitative-quantitative phenolic profile. At the same time, fruitiness is primarily linked to some molecules originating from the so-called lipoxygenase pathway, such as aroma-active esters [37]. EU Regulation for the EVOO category requires a median value of 0.0 for any negative attribute and a median value above 0.0 for fruitiness [8]. These requirements were met by all the EVOO samples investigated in the present study. The results have shown a higher intensity of the three attributes for EVOO samples from the Apulian cultivars than for the other investigated EVOO. In particular, EVOO from Coratina stood out for bitterness and pungency, and Bella di Cerignola for fruitiness (Table 1). Among Umbrian cultivars, the Frantoio EVOO sample and one Dolce Agogia sample (4DA) had higher scores than the others (*p* < 0.05).

Regarding the above-mentioned dependence of bitterness and pungency sensory attributes on the phenolic compounds, the results of this study showed acceptable correlations between the total phenolic content, measured by HPLC analysis, and bitterness and pungency (Table S2), with correlation coefficients of 0.61 and 0.53. It can also be observed that the correlation degree increased when the 9BC EVOO sample was excluded, reaching correlation coefficients of 0.78 and 0.73 (Table S2). These results confirm the correlation between some EVOO positive sensory attributes and the phenolic content, even though a small sample size was studied.

### 2.3. Oxidative Stability Index and Antioxidant Content

**Figure 6:**
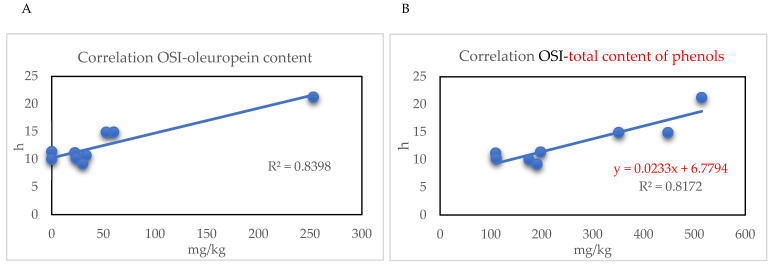
Correlation between oxidative stability index (OSI) and oleuropein content in all investigated EVOO samples (**A**), and between OSI and total content of phenolic compounds for the investigated EVOO samples, except Peranzana (**B**).

In this study, EVOO samples from different cultivars have also been subjected to the measurement of the oxidative stability index, a very useful parameter that best estimates the oils susceptibility to oxidative processes. It is strongly linked to the sample chemical composition, in particular to the content of antioxidant compounds, such as alpha-tocopherol and phenolic molecules. It is well known that oxidation processes, based on two different chemical mechanisms, autoxidation and photosensitized oxidation, have strong repercussions on palatability, nutritional quality, and toxicity of edible oils. Even if EVOO oxidative quality is higher than other vegetable oils due to the characteristic fatty acid composition (high oleic percentage content) and the presence of minor components with marked antioxidant activity [38], many efforts have been made to understand the relationship between EVOO stability and its chemical composition. In general, it should not be overlooked that the cultivar, the olive oil mereological category, and the extraction and conservation systems all decisively influence the antioxidant content and therefore also the oil stability and shelf life. The factors influencing the content of the antioxidant molecules have been extensively investigated, focusing particularly on the phenolic fraction [39] and the influence of technological variables on phenolic content and oxidative stability. The results obtained for OSI, expressed as oxidation induction time (h), indicated values between 9.19 and 21.24 h, with the highest stability obtained for Coratina and the lowest for Bella di Cerignola. Regarding the relationship between OSI and antioxidant compounds, the results showed an acceptable correlation degree between OSI and alpha-tocopherol content (correlation coefficient = 0.5216), excluding the Coratina sample. Regarding the relationship between OSI (Oxidative Stability Index) and phenolic compounds, interestingly, the best correlation was observed with oleuropein (R2 = 0.8398) (Figure 6A). When the total content of phenolic compounds was considered, the correlation with OSI was very high (R2 = 0.8172), excluding the Peranzana EVOO sample (Figure 6B).

It is widely known that phenols act as radical scavengers, able to donate an electron or hydrogen atom to a free radical, forming a stable molecule, thus interrupting the chain propagation of oxidation. A review from Bendini et al. (2007) reported several studies showing that oleuropein and its derivatives have higher antioxidant capacity than vitamin E, butylated hydroxytoluene (BHT), and other synthetic antioxidants [40]. The pronounced antioxidant activity of hydrophilic phenols in olive oil has been explained as the “polar paradox”, indicating that polar antioxidants are more effective in non-polar lipids [40]. Regarding the hydroxytyrosol content, a low correlation with the oxidative stability has been reported [41], as also observed in this study (R2 < 0.1). This occurrence has been ascribed to its trend to increase during storage, due to the hydrolysis of complex secoiridoid molecules.

### 2.4. Evaluation of Changes in the Content of EVOO Bioactive Compounds During Storage

**Figure 7:**
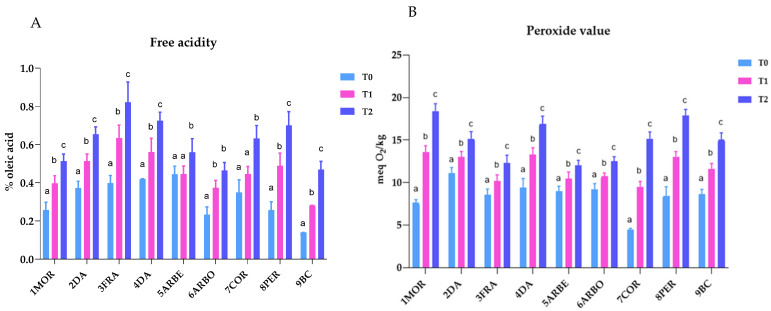
Bar graph showing the arithmetic mean and standard deviation of the free acidity (**A**) and peroxide value (**B**) of all the EVOO cultivars at the three sampling times. T0, before storage at r.t.; T1, after 2 months of storage at r.t.; T2, after 4 months of storage at r.t. Different superscript letters above the bars indicate significantly different values among storage times (Tukey’s test, *p* ˂ 0.05).

The last aim of the research was the evaluation of the modification of EVOO composition during storage for two and four storage months at room temperature (T1 and T2, respectively). In the following Figure 7, the trend of values of free acidity (A) and peroxide value (B) can be observed. The results showed that both parameters increased during storage, indicating that hydrolytic and oxidative processes occurred. For each cultivar, significant differences have been observed (*p* < 0.05) for both parameters, except for the free acidity of the following pairs: Arbequina T0-T1, Arbosana T1-T2, and Coratina T0-T1.

**Figure 8:**
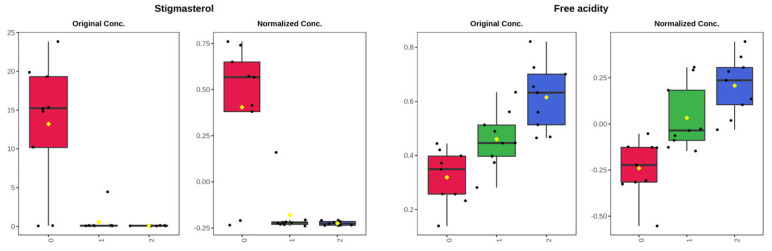

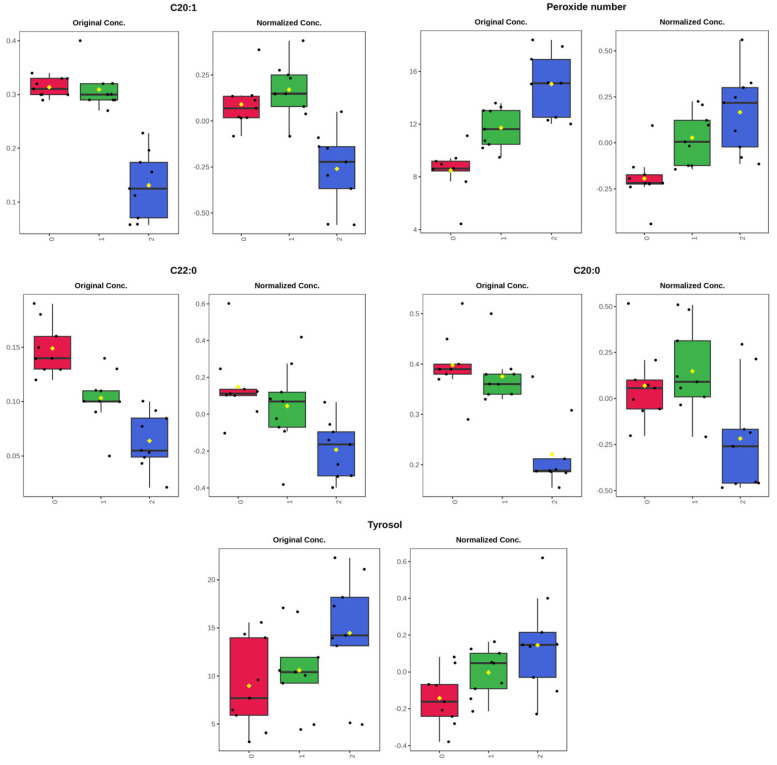
One-way ANOVA, box plots of the seven significant variables. 0, 1, and 2 groups correspond, respectively, to T0, before storage at r.t.; T1, after 2 months of storage at r.t.; T2, after 4 months of storage at r.t.

To evaluate the impact of storage on metabolite profiling, and thus on the nutritional and nutraceutical properties of EVOO, the compositional parameters of the samples from all the investigated cultivars, at the three storage times, were processed by multivariate statistical analysis in the MetaboAnalyst 6.0 platform (https://www.metaboanalyst.ca/MetaboAnalyst/ModuleView.xhtml, accessed on 30 April 2025). Data preprocessing involved sample normalization: median, data transformation: square root transformation, and data scaling: range scaling. Before data processing by PCA and PLS-DA, one-way ANOVA and post-hoc tests were performed. The results showed seven significant variables: stigmasterol, free acidity, C20:1, peroxide value, C22:0, C20:0, and tyrosol. Figure 8 shows the box plots with the original and normalized concentrations of the significant variables at the three sampling times (T0, before storage; T1, two-month storage; T2, four-month storage). It is possible to observe that stigmasterol underwent a drastic reduction already at T1 sampling, while, as commented above (Figure 7), the variables free acidity and peroxide value progressively increased during storage. Interestingly, some minor fatty acids were different in the three sampling groups, C20:1, C22:0, and C20:0, with a decreasing trend during storage. Differently, tyrosol underwent an increase from T0 to T2 sampling time, and this trend can be explained by the occurrence of hydrolysis reactions on secoiridoid molecules, as already mentioned above for hydroxytyrosol. There is much scientific evidence regarding the changes affecting the phenolic fraction due to secoiridoid degradation during storage. For example, Castillo-Luna and colleagues (2021) reported that hydroxytyrosol and oleocanthalic acid increased significantly in aged EVOO, so much so that these molecules could be considered markers of olive oil aging [16].

The results of the correlation analysis of the variables in the three sampling times (Figure S2) show several positive correlations, among which the highest (coefficient > 0.900) were observed for the following pairs: C18:3 n3-C18:0, C17:1-C17:0, C18:0-β-sitosterol, and C18:1 n9+n7-C16:0.

Figure S2 also indicates that some variables were negatively correlated, such as the pairs hydroxytyrosol-pinoresinol, oleuropein-C16:1, and hydroxytyrosol-C17:0, which showed the lowest correlation coefficient (lower than −0.630).

**Figure 9:**
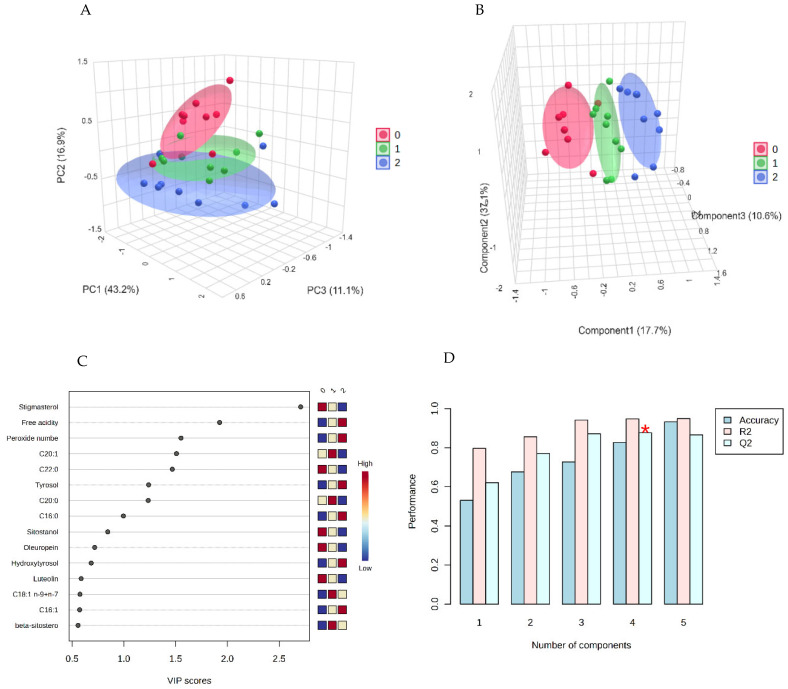
EVOO metabolite processing, T0-T1-T2. PCA: 3D score plot (**A**); PLS-DA: 3D score plot (**B**), variable importance in projection (**C**), cross-validation (**D**). 0, 1, and 2 groups correspond, respectively, to T0, before storage at r.t.; T1, after 2 months of storage at r.t.; T2, after 4 months of storage at r.t.

The results of EVOO compositional data processing at the three sampling times by PCA and PLS-DA are reported in Figure 9. PCA 3D score plot (Figure 9A) shows a partial overlapping of the sample groups related to different sampling times, as it is also possible to observe in the PCA pairwise score plots for the five principal components shown in Figure S3. However, PLS-DA, a supervised classification method that uses group labels to maximize the separation between groups, gave better separation of sample groups, as it is possible to observe in Figure 9B, where the 3D score plot of the first three discriminant functions is shown. The VIP scores obtained from the PLS-DA model, shown in Figure 9C, were used for the identification of significant metabolites responsible for cluster separation in the supervised model. The compounds with VIP score > 1, considered as differential metabolites in the study, were: stigmasterol, free acidity, peroxide value, C20:1, C22:0, tyrosol, and C20:0. Further, PLS-DA model was validated through the leave-one-out cross-validation approach and the quality evaluated on the basis of three measures (Accuracy, R2, Q2). Figure 9D, showing the cross-validation results by bar plots, proves the model validity, with the red asterisk indicating the best classifier (R2 = 0.86, Q2 = 0.94).

In recent decades, there has been an increase in the use of chemometric methods and multivariate statistical models in the food sciences. Regarding olive oil, it was not fully explored until recently. The study of Esposto et al. (2021) is an example of the combination of analytical techniques and a chemometric approach [42]. The authors investigated the changes of EVOO samples during storage in different packaging systems and processed the data by Principal Component Analyses (PCA) and orthogonal partial least squares regression discriminant analysis (O-PLS-DA). Spectroscopic techniques have also been used to monitor EVOO quality during storage, with the aid of multivariate statistical approaches for data processing [43].

The obtained results confirm that metabolite analysis reveals insight into the changes of EVOO composition and correlated properties during storage. Moreover, chemometric analysis provided important benefits, such as evaluating the significant parameters responsible for the changes during storage and presenting the results using scatter plots, indicating sample clustering or discrimination.

## 3. Materials and Methods

### 3.1. Reagents and Solvents

The reagents used for titrations, including sodium hydroxide, potassium iodide, acetic acid, chloroform, and sodium thiosulfate, were purchased from Merck Life Science (Milan, Italy). The analytical standards of α-tocopherol, tyrosol, hydroxytyrosol, syringic acid, oleuropein, luteolin, pinoresinol, squalene, 5α-cholestan-3β-ol, stigmasterol, sitostanol, and β-sitosterol were also obtained from Merck Life Science, while standards of oleacein and oleocanthal were purchased from PhytoLab (Vestenbergsgreuth, Germany). Supelco 37 FAME standard mix was from Supelco (Bellefonte, PA, USA). All the employed solvents were of analytical grade or HPLC-grade and were purchased from Carlo Erba (Milan, Italy).

### 3.2. Samples and Storage Conditions

Monovarietal extra-virgin olive oils, produced in Year 2023, were provided by several certified industrial mills. In particular, Gaudenzi Mill, located in Trevi (Perugia) provided the samples Moraiolo (1MOR) and Dolce Agogia (2DA), the Centumbrie company, located in Capanne (Perugia) provided the samples Frantoio (3FRA) and Dolce Agogia (4DA), the Azienda Agraria Buccelletti, located in Castiglione del Lago (Perugia) provided the samples Arbequina (5ARBE) and Arbosana (6ARBO) and the company Azienda Agricola Corleto, located in Ascoli Satriano (Foggia) provided the samples Coratina (7COR), Peranzana (8PER) and Bella di Cerignola (9BC). EVOO samples were divided into three groups: Umbrian cultivars (1MOR, 2DA, 3FRA, 4DA), Spanish cultivars (5ARBE, 6ARBO), and Apulian ones (7COR, 8PER, 9BC). Following filtration, the oils were stored in 250 mL amber glass bottles, sealed with hermetic caps, and maintained in the dark at room temperature. The storage protocol included three treatment periods, T0, T1, and T2, spaced two months apart. After each time point, bottles were resealed under standard conditions and returned to storage for the subsequent period. No special procedures were employed to remove oxygen, thus mimicking the typical gradual consumption and progressive oxidation occurring during prolonged storage and consumer use.

### 3.3. Sensory Profile Evaluation

The sensory attributes of the oil samples were assessed by the tasting panel of the Società Agricola Aprol Umbria organization, following the International Olive Council (IOC) method for virgin olive oil sensory evaluation [44], with all recommendations duly applied. Each olfactory and gustatory sensation was rated on a scale from 0 to 10. Samples were presented blindly to the assessors. The tasting session began with a calibration exercise led by the panel leader, who provided different oils to evaluate the response of each panel member. Following calibration, the leader, in a separate room, assigned a unique number to each oil replica and sequentially served the panelists, with each session including up to six oils per round (two rounds in total, covering eleven oils). The panel leader subsequently reviewed the results, calculating the average scores and verifying that all ratings fell within the acceptable range. In cases where scores were out of range, a new calibration was initiated, and the sample was renumbered and re-evaluated. Both positive attributes (fruitiness, bitterness, pungency) and negative characteristics (such as defects including moldy/muddy, musty/humid/earthy, winey/vinegary/acidic/sour, rancidity, among others) were evaluated. The panel leader compiled the individual notes, and statistical evaluation was performed by calculating the median for each parameter. This systematic approach provided sequential insights into the evolving sensory characteristics of the samples.

### 3.4. Determination of Peroxide Value

The values were obtained using the method described by the International Olive Council [45] with some modifications. Three grams of oil were weighed into a 250 mL container. Then, 25 mL of a pre-prepared acetic acid–chloroform solution (3:2 ratio) were added, followed by 1 mL of saturated potassium iodide solution. The mixture remained in the dark for 5 min. After the dark incubation, 75 mL of distilled water and 4–5 drops of starch solution were incorporated. The sample was titrated with 0.01 N sodium thiosulfate until a color change was observed, and the titrant volume was recorded.

### 3.5. Determination of Free Fatty Acids

The results were achieved by adapting the protocol established by the International Olive Council (IOC) [46], with specific modifications. EVOO samples (2 g) were weighed into a 250 mL flask, then a previously standardized ethanol/ethyl ether (1:1) mixture (50 mL) containing phenolphthalein was added. The oil mixture was then titrated with NaOH (0.1 N), and the volume of titrant used was measured.

### 3.6. Determination of α-Tocopherol

α-Tocopherol was analyzed using a modified HPLC method based on Tura et al., 2007 [47]. A 0.15 g olive oil sample was dissolved in 5 mL of hexane and homogenized by rotary shaking. The solvent was evaporated under nitrogen, and the residue was re-dissolved in 1 mL of isopropanol. HPLC analysis was performed on an Agilent 1100 series HPLC-DAD system (Agilent Technologies, Inc., Santa Clara, CA, USA) with a Kinetex 2.6 µm, 100 × 4.6 mm, Phenyl-Hexyl 100 Å column (Phenomenex, Torrance, CA, USA). The mobile phase consisted of water (Eluent A) and acetonitrile (Eluent B), with a gradient profile: 10:90 (A/B) for 6 min, changing to 100:0 from 6 to 10 min, and 90:10 from 10 to 15 min. The flow rate was 1.8 mL/min, and the total run time was 15 min. The injection volume was 5 µL. Detection and quantification were carried out at 292 nm. A calibration curve was established using α-tocopherol standard. Four standard solutions were used at the following concentrations: 45.8, 22.9, 11.45, and 4.58 mg/L.

### 3.7. Determination of Phenolic Compounds

The phenolic compounds in olive oil were extracted using a modified version of the method described by the International Olive Council (IOC) [48], with some adjustments. EVOO sample (2 g) was dissolved in 5 mL of an 80:20 methanol/water mixture, followed by 700 µL of an internal standard solution containing syringic acid. The samples were vortexed for 1 min and then centrifuged at 5000 rpm for 25 min at 4 °C. The resulting supernatant was injected into the HPLC-DAD system. The same apparatus and column, described in the previous 3.6 paragraph, were used. The mobile phase A consisted of acidic water (H_2_O with 0.2% phosphoric acid), while mobile phase B was methanol. The initial solvent composition was 95% A-5% B, and it changed to 100% B after 37 min, with a flow rate of 1 mL/min. The data acquisition was obtained at the wavelength set at 280 nm. The quantification of phenolic compounds was carried out using the external standard method, with calibration curves established for each of the investigated compounds. To identify and quantify phenols, the following compounds (concentrations of the relative standard solutions) were used as standards: hydroxytyrosol (8.3, 33, 66.7, 100, 158.3 mg/L), tyrosol (8.4, 33, 67.3, 101, 159 mg/L), oleacein (18, 36, 54, 85 mg/L), oleuropein (8.8, 35, 70, 105, 166 mg/L), oleocanthal (8.9, 36, 71, 107, 169 mg/L), pinoresinol (9.1, 36, 72, 109, 173 mg/L), and luteolin (8.3, 33, 67, 100, 158 mg/L).

### 3.8. Determination of Fatty Acid Methyl Esters (FAMEs) Profiles

Approximately 150 µL of olive oil was weighed and mixed with 2 mL of hexane. The transesterification was carried out by adding 200 µL of potassium hydroxide (2 M) solution in methanol and maintaining for 30 s at room temperature under vigorous shaking, then the hexane phase containing FAMEs was analyzed according to the method described by IOC [49]. The analysis was performed using a Varian CP3800 gas chromatograph (Varian, Walnut Creek, CA), equipped with a flame ionization detector (GC-FID), and a ZB-WAX capillary column (60 m × 0.25 mm i.d., 0.25 µm film thickness) composed of polyethylene glycol (Phenomenex, Torrance, CA, USA). Helium was used as the carrier gas, with a column flow rate of 1.5 mL/min and a split ratio of 1:100. The oven temperature program was as follows: the temperature was initially held at 140 °C for 2 min, then increased from 140 °C to 240 °C at a rate of 4 °C/min, followed by a 15-min hold at 240 °C, with a total run of 42 min. The identification of FAMEs was carried out by comparing their retention times with those of the Supelco 37 Component FAME Mix.

### 3.9. Analysis of Squalene and Sterols

Approximately 0.2 g of each oil sample was transferred into a 10 mL propylene tube, followed by the addition of 0.2 mL of an internal standard solution (5α-cholestan-3β-ol, 1000 ppm) in hexane. For the alkaline hydrolysis step, 2 mL of a 2% KOH solution was added, and the tubes were then incubated in a water bath at 80 °C for 15 min. The unsaponifiable components were extracted by vortexing the mixture with 1 mL of hexane and 1.5 mL of a 1% NaCl solution. The upper hexane layer was carefully transferred to 2 mL glass vials, and the samples were stored at −20 °C, with analysis performed within 24 h of preparation. The unsaponifiable fraction was directly analyzed by gas chromatography (GC) without a preceding thin-layer chromatography separation, a method that has been widely adopted for quantifying sterols and squalene in olive fruit and olive oil [50,51]. The chromatographic analysis was conducted using a GC-FID system fitted with a ZB-5HT Inferno capillary column (15 m × 0.32 mm × 0.10 μm, Phenomenex, Torrance, CA, USA). Helium served as the carrier gas at a flow rate of 1.5 mL/min, operating under a split ratio of 1:100. The oven temperature program started with an initial hold of 0.5 min at 150 °C, then increased from 150 °C to 240 °C at a rate of 8 °C/min, and further increased from 240 °C to 370 °C at 25 °C/min, holding at 370 °C for 5 min. The injector and detector (FID) temperatures were set at 320 °C and 350 °C, respectively. For the quantification of squalene and sterols, external standards were used (concentrations of the standard solutions): squalene (3989.5, 2979.5, 1969.5, 1010, and 505 mg/L); stigmasterol (70.2, 37.8, 10.8, and 1.08 mg/L); β-sitosterol (748, 393, 187, 93.5, and 46.8 mg/L); β-sitostanol (69.6, 40.6, 11.6, and 1.16 mg/L). Campesterol was quantified using the internal standard method.

### 3.10. Determination of Oxidative Stability Index (OSI)

A Metrohm Rancimat Model 743 (Metrohm, Herisau, Switzerland) was employed to assess the Oxidative Stability Index (OSI) of oil samples, as detailed in Metrohm Application Bulletin, 1993 [52]. In this procedure, 3 g of olive oil was placed in a reaction vessel situated in an electrically heated block maintained at 120 °C. A stream of filtered and dried air, flowing at 20 L/h, was bubbled through the olive oil. The effluent air, containing volatile compounds produced during oxidation, was then directed into a measuring vessel holding 60 mL of distilled water, where the conductivity was automatically recorded as oxidation progressed.

### 3.11. Statistical Analysis

All analyses were performed in triplicate (n = 3), with results expressed as the mean ± standard deviation (SD). Statistical significance was assessed using one-way ANOVA followed by Tukey’s HSD (honestly significant difference) post hoc test. The assumptions of normality and homoscedasticity of the data subjected to ANOVA were checked with the visual examination of the residuals against fitted values. A *p*-value less than 0.05 was considered statistically significant. GraphPad PRISM (version 9.3.1, GraphPad Software, Boston, MA, USA) and Microsoft Excel (version 16.91, Microsoft, Redmond, WA, USA) for Windows were used for statistical analysis and graph generation.

The results of metabolite contents were processed by multivariate analysis on the autoscaled dataset in the MetaboAnalyst 6.0 platform (http://www.metaboanalyst.ca/faces/home.xhtml, accessed on 12 May 2025). In particular, PCA and PLS-DA were applied. Two different datasets were used: the metabolite content at T0 to highlight compositional differences among cultivars, and the metabolite content at the three sampling times (T0, T1, and T2) to evaluate the impact of storage on EVOO chemical/nutritional properties. Figures S4 and S5 indicate the results of metabolite processing (sample normalization, data transformation, and data scaling) for the compositional differences among cultivars at T0 and among the different sampling times, respectively.

## 4. Conclusions

Given the increasing global demand for high-quality olive oil, monitoring its nutraceutical properties and stability over storage through robust chemical analysis protocols is paramount in ensuring product quality, safety, and consumer satisfaction. The results of this study allow for a deep characterization of olive oil from different cultivars, some of which were less investigated. As the first systematic study on the nutraceutical properties of the Bella di Cerignola cultivar (traditionally used for table olives), this work contributes to increasing knowledge of the chemical-nutritional and sensorial properties and stability of different EVOO cultivars. This paper successfully demonstrated the potential of using the chemical parameters, particularly the content of bioactive metabolites, to classify and differentiate among olive oils obtained from different cultivars grown in Italy. By applying PCA and PLS-DA, the results allowed for the discrimination of the olive oils according to cultivar first, and then to storage time, underscoring the validity of the approach despite the relatively small sample size. Given the importance of EVOO deep characterization, both in terms of cultivars and storage stability, research must continue to obtain the most reliable and strong evidence for ensuring olive oil quality. The chemical profiling of bioactive compounds is becoming even more important today, given their interesting implications for consumer health.

## Figures and Tables

**Table 1:** Results of the positive sensory attributes of EVOO cultivars.

	Fruitiness	Bitterness	Pungency
**1MOR**	3.00 ± 0.52 ^a^	1.60 ± 0.15 ^a^	2.74 ± 0.44 ^a,b^
**2DA**	1.88 ± 0.23 ^b^	1.58 ± 0.37 ^a^	1.74 ± 0.35 ^a^
**3FRA**	4.68 ± 0.66 ^c^	4.31 ± 0.78 ^b,c^	3.88 ± 1.30 ^b,c^
**4DA**	3.79 ± 0.45 ^a,d^	3.94 ± 1.11 ^b,d^	4.14 ± 0.69 ^c^
**5ARBE**	1.99 ± 0.36 ^b^	1.71 ± 0.38 ^a^	1.91 ± 0.67 ^a^
**6ARBO**	3.15 ± 0.75 ^a^	2.21 ± 0.54 ^a^	2.33 ± 0.59 ^a^
**7COR**	4.84 ± 0.66 ^c^	5.08 ± 0.32 ^c^	4.83 ± 0.93 ^c,d^
**8PER**	4.26 ± 0.63 ^c,d^	4.19 ± 0.58 ^b,d^	4.03 ± 0.77 ^c^
**9BC**	5.05 ± 0.57 ^c^	4.11 ± 0.53 ^b,d^	4.18 ± 0.62 ^d^

Data are presented as mean and standard deviation (SD) (n = 8). Values with different superscripts within a column are significantly different, *p* < 0.05.

## Data Availability

Data are contained within the article.

## Supplementary Materials

The following supporting information can be downloaded at: https://www.mdpi.com/article/10.3390/molecules30153143/s1, Table S1. Analysis of Variance, T0 EVOO samples, Feature Details Table; Table S2. Linear regression showing the correlation degree between the total content of phenolic compounds (mg/kg) and bitterness score, and the total content of phenolic compounds (mg/kg) and pungency score for the investigated samples. The same correlations, excluding the 9BC sample, are shown; Figure S1. EVOO metabolite processing, T0. Correlation heatmap, features, T0 EVOO samples; Figure S2. EVOO metabolite processing, T0-T1-T2. Correlation heatmap, features of EVOO samples at the three sampling times; Figure S3. EVOO metabolite processing, T0-T1-T2. PCA pairwise score plots for the five Principal Components; Figure S4: EVOO metabolite processing, T0, Umbrian-Spanish-Apulian cultivars. Sample normalization: median; data transformation: log transformation; data scaling: mean centering; Figure S5. EVOO metabolite processing, T0-T1-T2. Sample normalization: median; data transformation: square root transformation; data scaling: range scaling.
