# Supplementary material for: Preliminary Evaluation of the Nutraceutical Properties in Monovarietal Extra-Virgin Olive Oils and Monitoring Their Stability During Storage
Source: Molecules. 2025 Jul 26;30(15):3143. doi: 10.3390/molecules30153143 (PMC12348949; doi:10.3390/molecules30153143)
Supplement: Supplementary file 1 [file molecules-30-03143-s001.zip › molecules-3722763-supplementary.pdf]

Preliminary Evaluation of the Nutraceutical Properties in Monovarietal Extra-Virgin Olive Oils and Monitoring their Stability during Storage

Lina Cossignani [1,*], Ornella Calderini [2], Antonello Marinotti [3], Emiliano Orrico [3], Andrea Domesi [3], Luisa Massaccesi [3], Mirko Cucina [3], Marina Bufacchi [3,*]

[1] Section of Food, Biochemical, Physiological and Nutritional Sciences, Department of Pharmaceutical Sciences, University of Perugia, Perugia, 06126, Italy
[2] Institute of Biosciences and Bioresources, National Research Council, 06128 Perugia, Italy; ornella.calderini@cnr.it
[3] Institute for Agricultural and Forest Systems in the Mediterranean, National Research Council, 06128 Perugia, Italy; antonellomarinotti@cnr.it (A.M.); emilianoorrico@cnr.it (E.O.); andrea.domesi@cnr.it (A.D.), luisa.masssaccesi@cnr.it (L.M.); mirko.cucina@cnr.it (M.C.)
* Correspondence: marina.bufacchi@cnr.it (M.B.); lina.cossignani@unipg.it (L.C.); Tel.: +39-075-5014538 (M.B.); +39-075-585-7959 (L.C.)

Table S1. Analysis of Variance, T0 EVOO samples, Feature Details Table

	f.value	p.value	-log10(p)	FDR	Post-hoc tests		
Stigmasterol	570.93	5.79E-17	20.237	1.56E-15	1-2	3 - 2	
Oleuropein	179.73	3.61E-11	14.442	4.88E-10	1-2	3 - 2	
Luteolin	35.409	6.92E-04	71.601	6.23E-04	2-1	2 - 3	
Campesterol	30.467	2.59E-03	65.865	1.75E-02	1-2	3 - 2	
Squalene	18.23	1.53E-02	4.815	8.27E-01	1-2	3 - 1	3 - 2
C17:1	12.472	0.000193	37.138	0.00087	2-1	2 - 3	
Sitostanol	95.759	0.000876	30.574	0.002958	1-2	3 - 2	
C16:1	9.575	0.000877	30.572	0.002958	2-1	2 - 3	
Pinoresinol	79.915	0.002188	2.66	0.006564	2-1	3 - 1	
Free acidity	78.118	0.002438	26.129	0.006583	1-3	2 - 3	
C17:0	70.117	0.003999	23.981	0.009815	2-1	2 - 3	
Peroxide number	6.433	0.005795	2.237	0.013038	1-3	2 - 3	
C20:0	50.483	0.014791	1.83	0.030721	2-1	3 - 1	

Table S2. Linear regression showing the correlation degree between total phenol content (mg/kg) and bitterness score, and total phenol content (mg/kg) and pungency score for the investigated samples. The same correlations, excluding the 9BC sample, are shown.

**Correlation**	**Regression equation**	**R^2**
Total phenol content *vs* bitterness	y = 0.0055x + 1.5014	0.6110
Total phenol content *vs* bitterness[a]	y = 0.0062x + 1.0891	0.7812
Total phenol content *vs* pungency	y = 0.0042x + 2.0001	0.5293
Total phenol content *vs* pungency[a]	y = 0.0048x + 1.628	0.7267

[a]Excluding sample 9BC

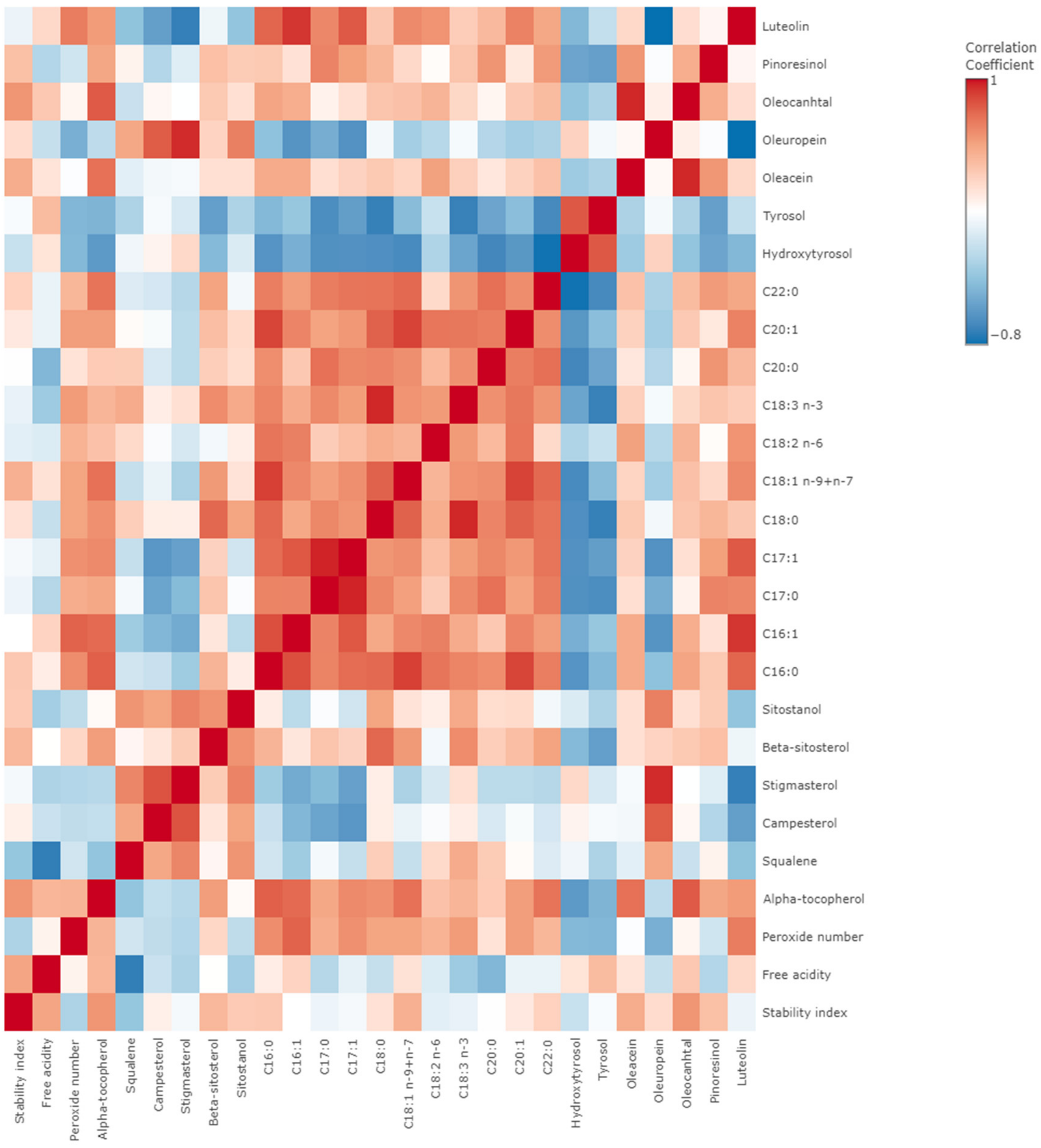

Figure S1. EVOO metabolite processing, T0. Correlation heatmap, features, T0 EVOO samples

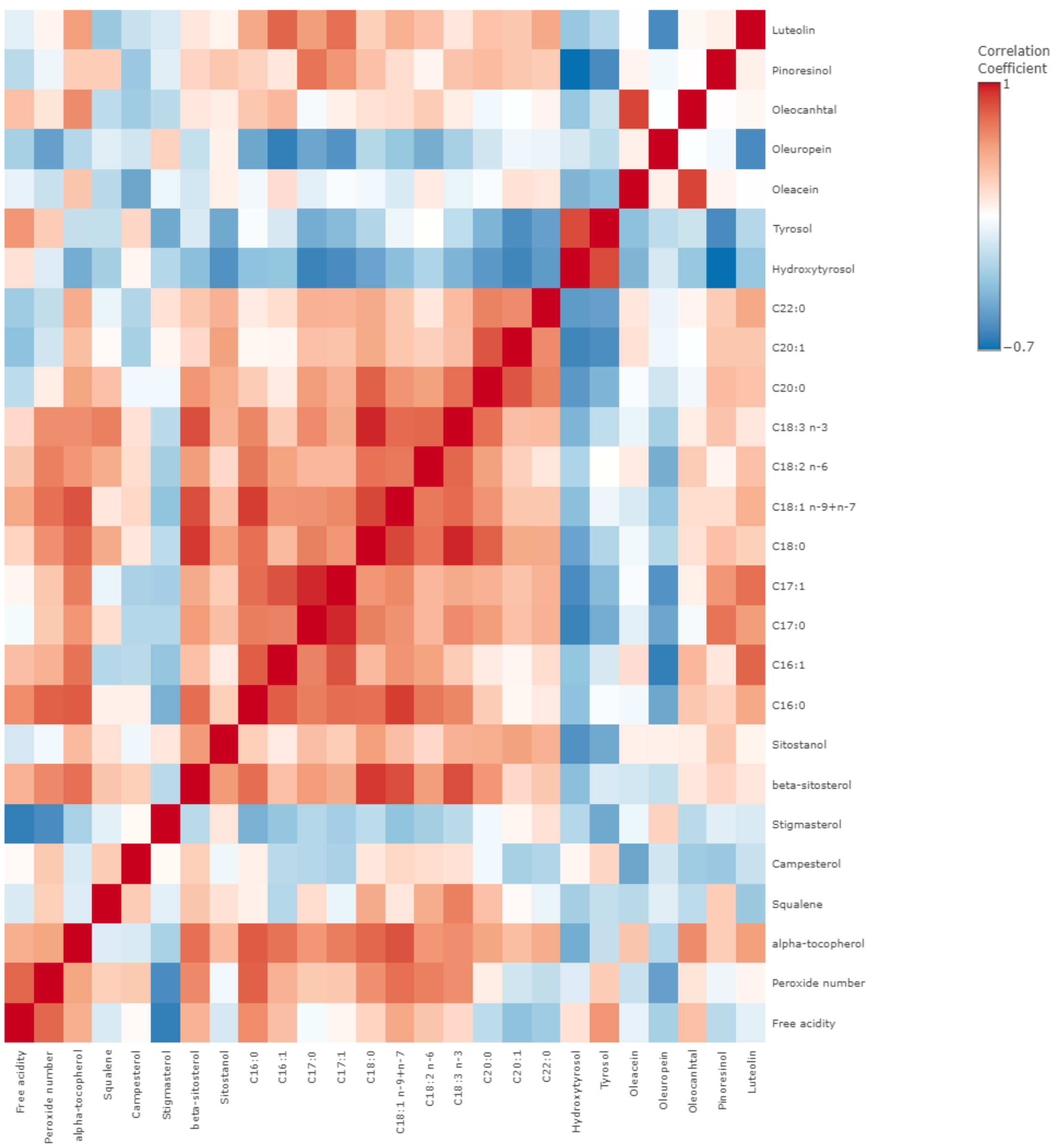

Figure S2. EVOO metabolite processing, T0-T1-T2. Correlation heatmap, features of EVOO samples at the three sampling times.

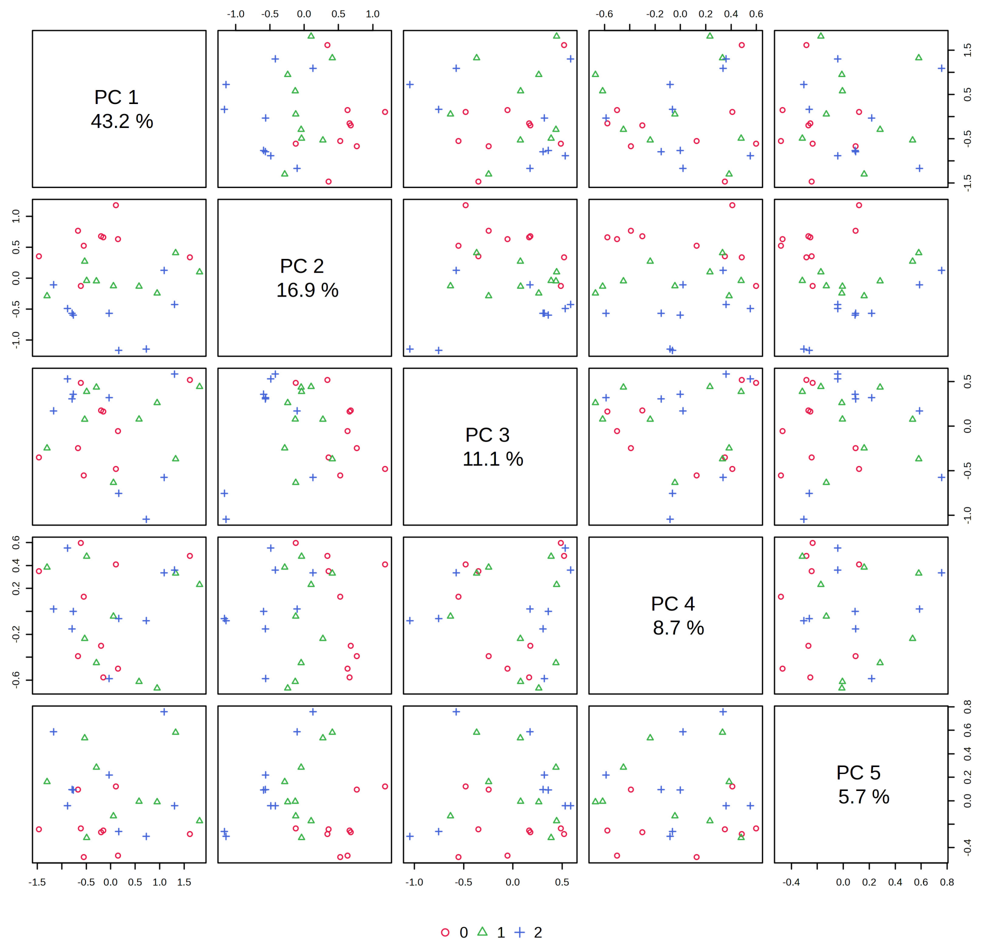

Figure S3. EVOO metabolite processing, T0-T1-T2. PCA pairwise score plots for the five Principal Components.

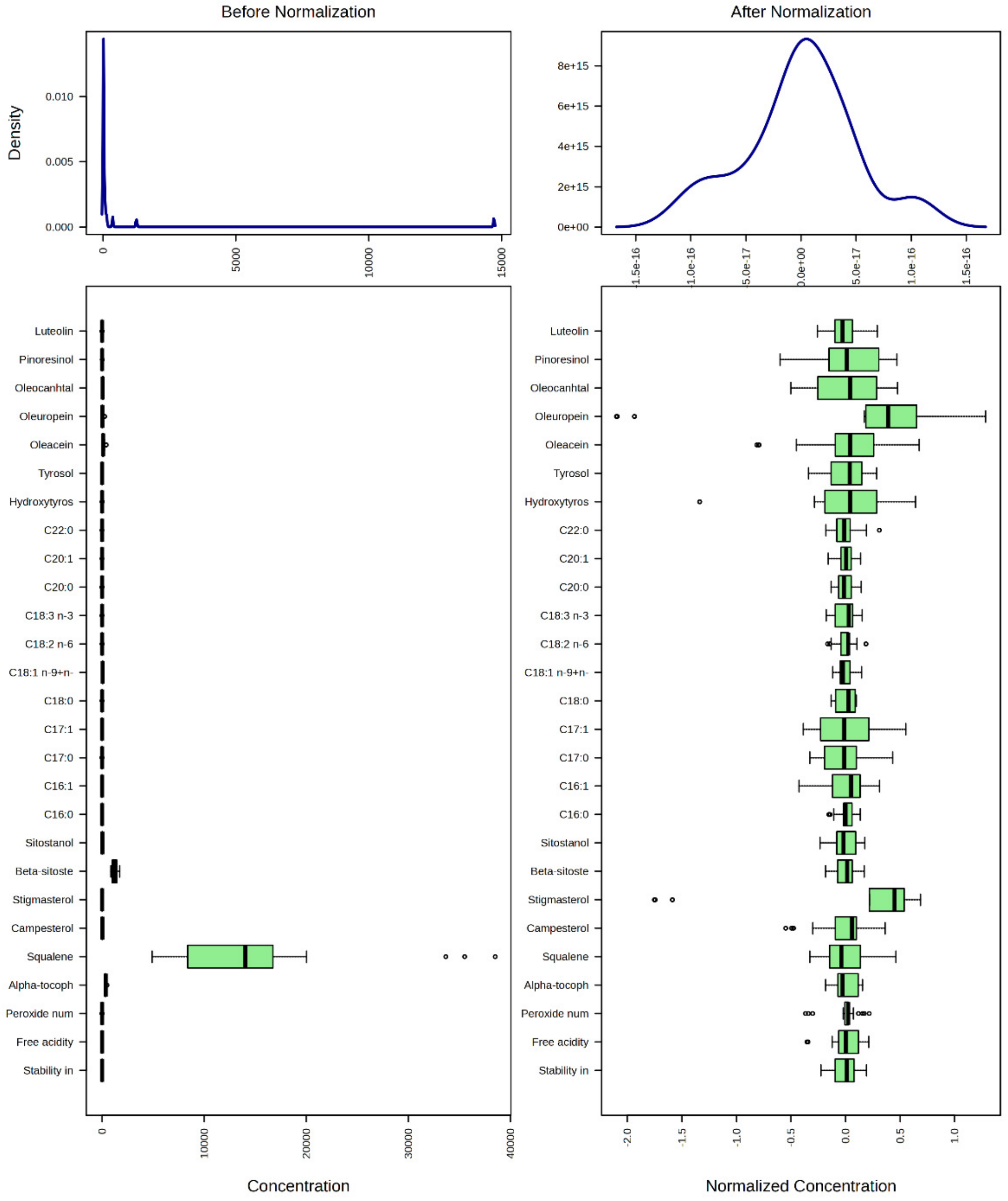

Figure S4. EVOO metabolite processing, T0, Umbrian-Spanish-Apulian cultivars. Sample normalization: median; data transformation: log transformation; data scaling: mean centering.

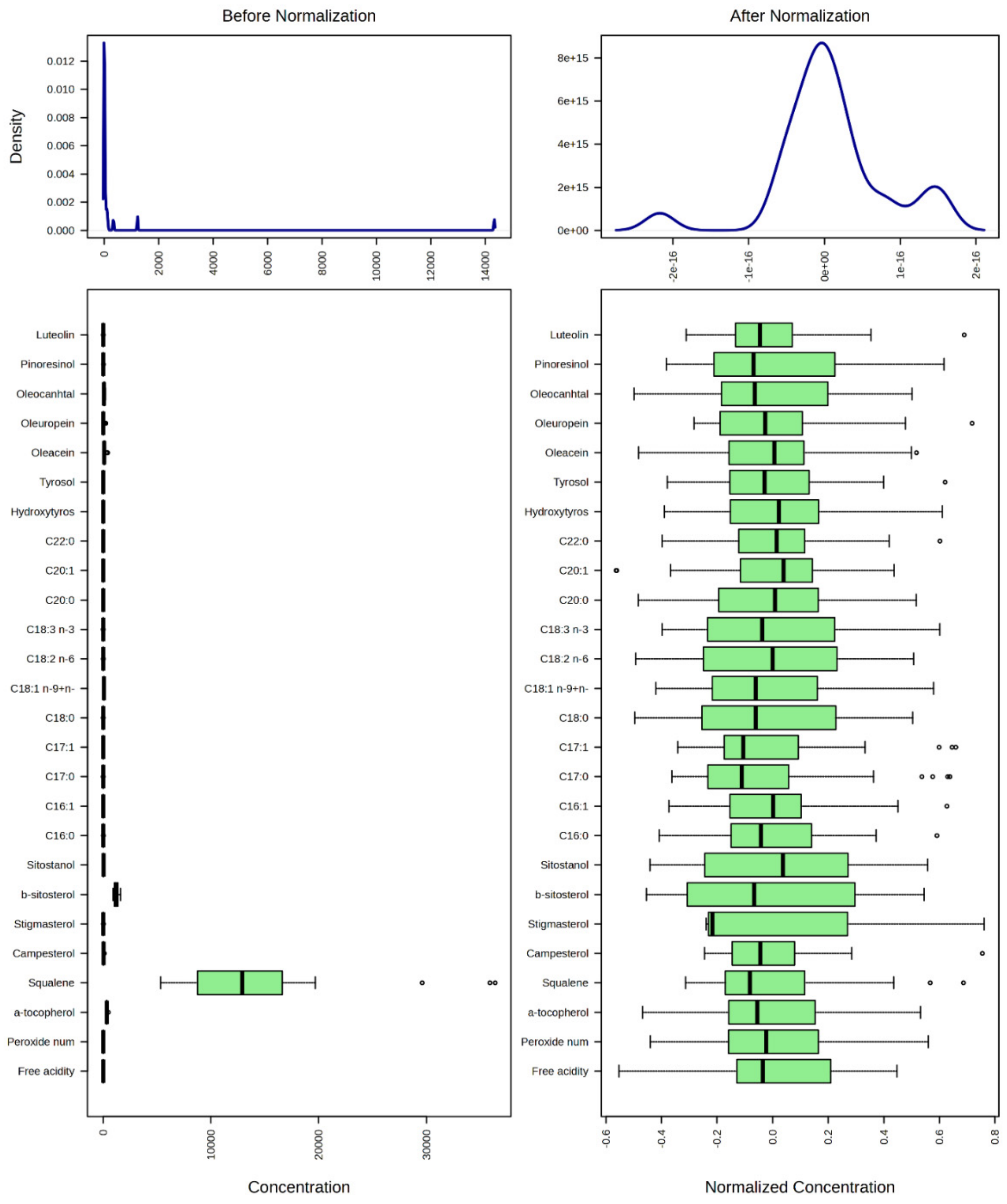

Figure S5. EVOO metabolite processing, T0-T1-T2. Sample normalization: median; data transformation: square root transformation; data scaling: range scaling.
